# Prevalence and Global Health Implications of Social Media in Direct-to-Consumer Drug Advertising

**DOI:** 10.2196/jmir.1775

**Published:** 2011-08-31

**Authors:** Bryan A Liang, Timothy K Mackey

**Affiliations:** ^1^Department of Anesthesiology, San Diego Center for Patient SafetySan Diego School of MedicineUniversity of CaliforniaSan Diego, CAUnited States; ^2^Institute of Health Law StudiesCalifornia Western School of LawSan Diego, CAUnited States; ^3^Joint Doctoral Program in Global HealthSan Diego State University-University of CaliforniaSan Diego, CAUnited States

**Keywords:** Illegal pharmacies, social media, pharmaceutical marketing, direct-to-consumer-advertising, internet pharmacies, global health, law, health policy

## Abstract

**Background:**

Direct-to-consumer advertising (DTCA), linked to inappropriate medication use and higher health care expenditures, is the fastest growing form of pharmaceutical marketing. DTCA is legal only in the United States and New Zealand. However, the advent of online interactive social media “Web 2.0” technologies—that is, eDTCA 2.0—may circumvent DTCA legal proscriptions.

**Objective:**

The purpose of this study was to assess the prevalence of DTCA of leading pharmaceutical company presence and drug product marketing in online interactive social media technologies (eDTCA 2.0).

**Methods:**

We conducted a descriptive study of the prevalence of eDTCA 2.0 marketing in the top 10 global pharmaceutical corporations and 10 highest grossing drugs of 2009.

**Results:**

All pharmaceutical companies reviewed (10/10, 100%) have a presence in eDTCA 2.0 on Facebook, Twitter/Friendster, sponsored blogs, and really simple syndication (RSS) feeds. In addition, 80% (8/10) have dedicated YouTube channels, and 80% (8/10) developed health care communication-related mobile applications. For reviewed drugs, 90% (9/10) have dedicated websites, 70% (7/10) have dedicated Facebook pages, 90% (9/10) have health communications-related Twitter and Friendster traffic, and 80% (8/10) have DTCA television advertisements on YouTube. We also found 90% (9/10) of these drugs had a *non*-corporate eDTCA 2.0 marketing presence by illegal online drug sellers.

**Conclusion:**

Pharmaceutical companies use eDTCA 2.0 to market themselves and their top-selling drugs. eDTCA 2.0 is also used by illicit online drug sellers. Regulators worldwide must take into account the current eDTCA 2.0 presence when attempting to reach policy and safety goals.

## Introduction

Pharmaceutical direct-to-consumer advertising (DTCA) is legal only in the United States and New Zealand among industrialized countries [1]. It is linked with inappropriate medication use, overutilization, and increased spending on expensive branded drugs, and it may endanger public health due to promotion of potentially dangerous products [2]. International DTCA legal proscriptions indicate that sovereign states have deemed DTCA risks to outweigh its benefits [3]. Yet it is the fastest growing form of marketing, rising 330% from 1996 to 2005 [2], with about US $4.3 billion 2009 expenditures [4], outpacing physician marketing and research and development spending over the past decade [5].

With the Internet’s rapid development, users have migrated from passive information sources, using read-only “Web 1.0” technology, to interactive, dynamic, and custom-built relationships, using “Web 2.0” technologies [6]. Along with this digital revolution, new potential DTCA marketing opportunities haven recently emerged that include Web 2.0 social networking sites and other interactive systems (“eDTCA 2.0”), which cross geopolitical borders [7]. The US Food and Drug Administration (FDA) has not issued guidelines on eDTCA 2.0 marketing, nor have regulators recognized eDTCA 2.0 and its potential global spillover.

With DTCA public health concerns, lack of specific US regulation, global DTCA legal prohibition, and the Internet’s extensive reach via social networking and other Web 2.0 technologies, we assessed global pharmaceutical company eDTCA 2.0 presence for potential marketing. Here, the focus was the corporate presence in eDTCA 2.0 social media for potential marketing, rather than the accuracy of DTCA claims, which has been found problematic elsewhere [5,8]. In addition, we wished to assess whether top-selling drugs have an established eDTCA 2.0 presence, potentially avoiding global DTCA prohibitions.

## Methods

We identified the 10 largest pharmaceutical companies and the top 10 grossing medicines worldwide in 2009 using IMS Health sales data, which tracks revenue by company and product [9].

Google searches were run to identify their eDTCA 2.0 presence. Important to note is that eDTCA 2.0 presence contains a mix of DTCA categories, including reminder advertisements that only contain information about a disease or condition and recommend health care provider consultation; help-seeking advertisements that include product name and therapeutic claims; and product-claim advertisements that only provide the product name [10]. The prevalence of social media marketing tools used by pharmaceutical corporations was determined by searching for dedicated corporate social media sites, Facebook pages, Twitter or Friendster accounts, blogs or really simple syndication (RSS) feeds, and YouTube channels, and whether corporations had developed and sold health-related mobile applications in the Apple iTunes store.

Dedicated corporate social media sites were defined as manufacturer-hosted websites launching multiple company social media marketing tools, including Facebook and Twitter or Friendster. Facebook is the leading social network interactive service connecting 500 million users [11]. Twitter, a popular US-dominant social media site with 106 million users [12], and Friendster, with a strong international presence, particularly in Asia, with 100 million users [13], are social networking and microblog sites using short Internet posts. Corporate blogs and RSS feeds are company-sponsored Web feed formats providing communication to users. Corporate YouTube channels were defined as company-sponsored sites with dedicated channels for its marketing videos. Corporate mobile application presence was defined as mobile applications identified by pharmaceutical company name and copyright for smartphones and other mobile technologies in the iTunes store. Foreign corporate subsidiaries and affiliates were also included in these searches. A diverse mix of eDTCA 2.0 categories occurs in this space, but the predominant category of DTCA is help-seeking advertisements.

In assessing eDTCA 2.0 prevalence for drugs, searches were conducted to determine whether each specific product had a dedicated website, product-specific Facebook pages, user-generated Twitter/Friendster postings, and YouTube-available DTCA. Dedicated product pages were identified as manufacturer websites that solely marketed the product. A corporate Facebook page where the profile was identified by product name and description was defined as a product-specific Facebook page. In determining whether products were the subject of Twitter/Friendster postings, all postings uploaded by all users that discussed health-related communications were reviewed for drug-specific discussions. YouTube-available DTCA was identified as any generated viewable video content upload of video-broadcasted DTCA relating to the specific drug. eDTCA 2.0 for drug products also includes a mix of DTCA types; however, the predominant category in this space is product-claim advertisements, which identify the product and the therapeutic claim along with safety and efficacy information.

Searches were conducted from September 2010 to December 2010 using the Google search engine. The search strategy for identifying dedicated social media marketing tools used by pharmaceutical companies included the following key word searches. For dedicated manufacturer-hosted social media sites the identified corporation name and the term “social media” were used for the search (eg, “Pfizer+social media”). For specific social media tools used by pharmaceutical companies, we used social media tool links from the identified manufacturer’s hosted social media website results and key word searches consisting of the identified corporation name and the social media tool type (eg, “Pfizer+Facebook”). In assessing eDTCA 2.0 presence for drug products, searches for dedicated product websites used the identified drug name and the key word “official website” (eg, “Lipitor+official website”). For product-specific social media tools we used the identified drug name and the social media tool type (eg, “Lipitor+Facebook”).

## Results

Table 1 summarizes the eDTCA 2.0 presence of the top 10 pharmaceutical corporations. Of these, 40% (4/10) had dedicated social media corporate websites linking to all the company’s other social media marketing tools. The world’s largest pharmaceutical corporation, Pfizer, had the most robust social media website, including links to YouTube, Facebook, and Twitter, as well as SlideShare, LinkedIn, Flickr, and blog resources. All (10/10, 100%) corporations had corporate Facebook pages, Twitter feeds, and some type of sponsored blogs or RSS feeds, while 80% (8/10) had dedicated YouTube channels and health care-related mobile applications.

**Table 1:** Top 10 pharmaceutical companies by sales and eDTCA 2.0^a^ presence

Company	Dedicated social media site	Facebook page	Twitter/Friendster	Sponsored blogs/RSS^b^	YouTube channel	Mobile applications
Pfizer	Yes	Yes	Yes	Yes	Yes	Yes
Merck & Co.	No	Yes	Yes	Yes	No	Yes
Novartis	No	Yes	Yes	Yes	Yes	Yes
Sanofi-Aventis	Yes	Yes	Yes	Yes	Yes	Yes
GlaxoSmithKline	No	Yes	Yes	Yes	Yes	Yes
AstraZeneca	Yes	Yes	Yes	Yes	Yes	Yes
Roche	Yes	Yes	Yes	Yes	Yes	Yes
Johnson & Johnson	No	Yes	Yes	Yes	Yes	Yes
Eli Lilly	No	Yes	Yes	Yes	No	No
Abbott	No	Yes	Yes	Yes	Yes	No

^a^ Direct-to-consumer advertising developed for interactive social media “Web 2.0” technologies.

^b^ Really simple syndication.

Table 2 summarizes eDTCA 2.0 for 10 blockbuster branded drugs. Of these, 90% (9/10) have dedicated product pages; 70% (7/10) have product-specific dedicated Facebook pages; 90% (9/10) have health communications-related Twitter/Friendster traffic; and 80% (8/10) have DTCA advertisements on YouTube.

**Table 2:** Top 10 grossing drugs and eDTCA 2.0^a^ presence

Drug	Dedicated product page	Facebook page	Twitter/Friendster	YouTube	eDTCA 2.0 online pharmacy link
Lipitor	Yes	Yes	Yes	Yes	Yes
Plavix	Yes	No	Yes	Yes	Yes
Nexium	Yes	Yes	Yes	Yes	Yes
Seretide	Yes	No	Yes	Yes	Yes
Seroquel	Yes	Yes	Yes	Yes	Yes
Enbrel	Yes	Yes	Yes	Yes	No
Remicade	Yes	Yes	Yes	No	No
Crestor	Yes	Yes	Yes	Yes	Yes
Zyprexa	No (taken down)	No	Yes	No	Yes
Humira	Yes	Yes	No	Yes	No

^a^ Direct-to-consumer advertising developed for interactive social media “Web 2.0” technologies.

During searches for drug-specific, corporate eDTCA 2.0 presence, we also observed an unexpected finding: use of eDTCA 2.0 pharmaceutical marketing by illicit online pharmacies. Illicit online pharmacies are websites or links to websites identified as marketing the sale of drug products without a prescription [14]. In addition, identified websites and links to websites did not meet the verification criteria under the National Association of Boards of Pharmacy Verified Internet Pharmacy Practice Sites (VIPPS) program [15]. These illicit online pharmacies have historically used online marketing tools such as search engine optimization and search engine marketing tools as an illicit form of DTCA [7]. When performing these searches, we discovered that 90% (9/10) had a noncorporate eDTCA 2.0 presence, either advertising the purchasing of drugs online without a prescription or linking directly to illegal online pharmacies promoting sales without a prescription. The search was expanded to the top 20 globally marketed DTCA products. The expanded search used The Nielsen Company data to identify the top 20 drugs brands by DTCA spending [16]. The trend remained, with 80% (16/20) of the top 20 products either advertising or linking to illegal online drug sellers using eDTCA 2.0 tools (Table 3). Most included graphical advertisements and direct links to illicit sellers. The prevalence of illegal online drug sellers on Facebook alone was 60% (6/10) for the top 10 drugs and 50% (10/20) for the top 20.

**Table 3:** Top 20 drugs by global sales and eDTCA 2.0^a^ link to illegal online drug sellers

Drug	Facebook page link	Twitter/Friendster link
Lipitor	Yes	Yes
Ablify	Yes	Yes
Advair Diskus	No	Yes
Cymbalta	Yes	Yes
Cialis	Yes	Yes
Lyrica	No	No
Plavix	Yes	Yes
Symbicort	No	Yes
Ambien CR	Yes	Yes
Crestor	No	Yes
Viagra	Yes	Yes
Pristiq	Yes	Yes
Flomax	No	Yes
Chantix	No	Yes
Yaz	Yes	Yes
Enbrel	No	No
Celebrex	Yes	Yes
Boniva	No	No
Spiriva	No	No
Caduet	Yes	Yes

^a^ Direct-to-consumer advertising developed for interactive social media “Web 2.0” technologies.

## Discussion

### Pharmaceutical Digital Drive

The Internet’s global expanse has led to significant patient use. The National Center for Health Statistics reported 51% of adults searched for health information on the Internet from January to June 2009 [17], and a Harris poll estimated 175 million adults use the Internet for health care information [18]. With hundreds of millions of users of social media and other interactive tools [19], this market cannot be ignored.

Pharmaceutical companies appear to agree, adapting and engaging eDTCA 2.0 technologies to promote themselves and their highest grossing drugs. With near universal adoption of the most popular social media marketing represented by Facebook, Twitter/Friendster, and RSS feeds and blogs, companies are firmly committed to eDTCA 2.0. As well, 80% of firms with YouTube-dedicated channels and mobile health applications indicate they are also investing in other eDTCA 2.0 tools, such as multimedia formats with videos and health communications-related software.

However, these results indicate that eDTCA 2.0 marketing may not be limited to where DTCA is permitted by law. eDTCA 2.0 sites such as GlaxoSmithKline’s blog site and AstraZeneca’s community Facebook page indicate they are “intended for US residents/customers only,” but appear to offer no access restrictions to non-US users [7]. Further, mobile applications such as those advertised on Apple’s iTunes media store are marketed by large pharmaceutical firms such as Pfizer, Novartis, and Roche, and target Canadian, French, and Korean audiences. Hence, eDTCA 2.0 marketing may occur outside the United States and New Zealand [7].

Estimates of 2009 total online DTCA spending (including eDTCA 2.0) are between US $117 million and $1 billion. Although these figures appear lower than traditional DTCA expenditures, these figures may both underestimate [16,20] and not reflect Internet advertising’s significantly lower costs and greater benefits in reaching advertising markets [21,22]. These benefits include flexibility in marketing blockbuster and niche therapies, ability to reach larger audiences and target specific patients, and better financial analytics of social media and marketing return on investment [23]. Further, the scope of eDTCA 2.0 efforts is still not well defined. For example, some estimates include banners, advertisement displays, and advertisement time on streaming videos [24] but fail to capture eDTCA 2.0 presence in sponsored links, advertisement works, website development and hosting, and other interactive social media tools that have proliferated for promoting drugs [16].

### Regulatory Challenges

For public health policymakers and patients, the Internet’s escalating use is of concern. First, pharmaceutical manufacturers’ eDTCA 2.0 development and presence may set back even further FDA efforts to effectively regulate DTCA [8]. Internationally, global DTCA prohibitions are being emasculated by these promotion efforts. eDTCA 2.0 technology has moved DTCA outside geopolitical regulatory boundaries into a system of potentially unfettered promotion, openly accessible to manufacturers and patients worldwide. Indeed, eDTCA 2.0 has spread DTCA to anyone, anywhere with an Internet connection.

This may serve as a stark warning for public health policy, given earlier work that concluded that prohibited television-based US DTCA transmitted to Canada increased prescriptions for tegaserod by 42% after it began—a drug that was later withdrawn from US and Canadian markets due to safety concerns [25]. These developments are also of public health concern because reform efforts in the United States and abroad have not recognized issues specifically relating to eDTCA 2.0 [5,8,26]. This places these regions at risk for higher pharmaceutical prices [27] and increased costs associated with excessive prescriptions [28].

In addition, assessments of online drug advertisements show suspect quality and overemphasis of potential benefits [8]. Second, the most-advertised drugs have been those with large patient markets and/or that treat chronic or long-term conditions, placing many, particularly vulnerable, patients at risk [24]. But further, this risk may be disproportionately increased because these newer drugs are most heavily marketed early in product life cycles—when they may not be adequately assessed for safety [29-31]. Indeed, in the United States, of the top 20 DTCA-advertised drugs, 90% (18/20) were subject to a black box warning, recall, or other safety notification (Table 4). The United States’s adverse experience relating to drug promotion may become a global health concern under eDTCA 2.0, particularly in developing countries with growing chronic disease burdens [32].

**Table 4:** Prevalence of safety issues for top 20 products advertised direct to consumers, 2009

Drug	Safety warning^a^
Lipitor	Subject to recall; other safety notification
Ablify	Black box warning; subject to recall
Advair Diskus	Black box warning
Cymbalta	Black box warning
Cialis	Other safety notification
Lyrica	None
Plavix	Black box warning; subject to recall; other safety notification
Symbicort	Black box warning; other safety notification
Ambien CR	Other safety notification
Crestor	Other safety notification
Viagra	Other safety notification
Pristiq	Black box warning; other safety notification
Flomax	Other safety notification
Chantix	Black box warning
Yaz	Black box warning; other safety notification
Enbrel	Black box warning; subject to recall; other safety notification
Celebrex	Black box warning
Boniva	Other safety notification
Spiriva	Subject to recall
Caduet	None

^a^ “Other safety warnings” include voluntary recalls, counterfeit warnings, warnings on contraindications, warnings about combining with other drugs, and other US Food and Drug Administration-issued alerts.

### Rogue eDTCA 2.0

Adding even more public health risks and worries is the discovery that rogue online pharmacies are already entrenched in eDTCA 2.0. The presence of illegal online pharmacies using interactive social media to sell illicit products is of great worry [7]. First, from a legal point of view, this eDTCA 2.0 presence represents another way it can undermine legal proscriptions (eg, prohibition of medication sales without a prescription, license, or oversight). But more important, avoidance of such legal proscriptions goes deeper as a safety risk, as patients may purchase drugs not approved or that are the subject of safety concerns (eg, Zyprexa).

Indeed, illegal online pharmacies have succeeded in selling tainted or fake drugs globally [14]. They have used search engine-sponsored links to sell illegally and circumvent search engine mandates for legitimacy verification [14]. But these illicit sellers are highly nimble; after investigations by us and others [14,32] showing lack of search engine oversight and continued illicit sales, Google, followed by Yahoo and Bing/Microsoft, adopted recommendations to use only VIPPS accreditation [33,34]. However, after this change, although eliminating sponsored link presence, illicit online pharmacies deftly moved to be listed in search engine shopping pages. Only after notification of this development by one of us (Andrew Kline, Senior Advisor, Intellectual Property Enforcement, Executive Office of the President, oral communication, October 8, 2010) to a government representative was this loophole closed. However, it now appears that illicit online drug sellers have infiltrated eDTCA 2.0, including Facebook with its hundreds of millions of users worldwide.

Such illicit presence and online purchasing of drugs are rife with patient risks. Counterfeit, diverted, and unregulated drugs are sold by these illegal vendors causing patient harm [14]. With drug supply globalization, risks associated with unregulated, non-VIPPS sellers are legion, with a host of public health and regulatory agencies warning about online drug sellers [14,35,36,37,38].

### Global Health Problem

The combination of eDTCA 2.0 presence by drug companies and by illicit drug sellers creates even greater patient risks than each alone, and a more urgent imperative for public action [7]. Global eDTCA 2.0 from whatever source, licit or illicit, may inappropriately increase demand, and then compound harm by permitting self-prescribing and purchasing from online drug sellers directly linked from sites such as Facebook. Hence, patient exposure to potentially risky drugs or drug forms is fueled by both unregulated eDTCA 2.0 corporate marketing and illicit drug sellers populating social media.

Regulatory priority for eDTCA 2.0 is needed for other reasons. The relatively overstated benefits associated with traditional DTCA [39,40] may drive a corporate shift to more efficient eDTCA 2.0 marketing. This is especially relevant given studies showing that traditional forms of DTCA such as television, magazines, and the radio may have only a minor impact on sales of DTCA drugs [40]. Governments hence, may wish to anticipate this potential upswing by responding with aggressive regulation. Theoretically, this would also capture illicit online drug sellers, although the ease by which they can recreate their presence thwarts any single solution to addressing this concern [14]. Further, because search engines drive consumers to content [6], eDTCA 2.0 interactive media forms receive higher traffic due to repeat use or links from other websites. They will therefore often appear higher in search results and may have a disproportionate patient impact [41]. eDTCA 2.0 may consequently result in pharmaceutical companies becoming the dominant source of health-related information, replacing health care professionals [39,42].

### Reform Considerations

Given limited recognition and inadequate regulation of eDTCA 2.0, and difficulty instituting a global ban due to US legal considerations [43], alternative approaches are warranted. We believe solutions must at least include eDTCA export prevention, eDTCA funding-source transparency, and patient safety integration into eDTCA communications.

In preventing eDTCA 2.0 illegal export, similar to disease outbreak surveillance and control, the United States should take an active role in ensuring that eDTCA 2.0 originating from US-based companies or information technology infrastructure is not transmitted across its borders [7]. Given that eDTCA is a public health concern, The United States, as the largest producer and eDTCA exporter, needs effective policy to proactively regulate DTCA as a global export.

To do this, the United States should require marketers, pharmaceutical manufacturers, Web content providers (such as Internet service providers and registrars), and social media sites to engage in active surveillance and block foreign internet protocol (IP) address holders or users from viewing eDTCA 2.0 content. Such efforts would be analogous to prior activities between a joint task force between the FDA and Federal Trade Commission to combat illegal Web activity involving the sale of fraudulent H1N1 supplements [44]. Enforcement would specifically include social media sites such as Facebook and Twitter. Further, illicit online pharmacy presence in the most popular social media forms poses a serious public health risk to both individuals searching for health-related information and users increasingly dependent on social networks for information. Social media service providers must recognize the serious implications of not actively policing this illicit content. Public–private partnerships in developing filtering software can be part of this effort, which can be disseminated to domestic and global social media entities and regulators [7].

Another key component is clear eDTCA 2.0 sponsorship identification. Patients must be appraised about online sources, including financial underwriting, of eDTCA 2.0 content [7]. Often reliability of eDTCA 2.0 cannot be determined because consumers cannot discern or lack information about which parties own, administer, or fund it [8]. This is especially problematic when pharmaceutical companies use third parties such as bloggers, “consumer opinion leaders,” or other employed individuals or companies [45] to promote products without disclosing financial ties [8]; moderated forums or sites that appear interactive but offer only one-sided communication; and “unbranded websites” without sponsorship disclosure [46]. The latter may garner considerable consumer attention, complement other social media, and be effective in promoting downstream revenue [46]. Such potential hidden drug marketing should be exposed, taking into account the vagaries of social network tools including short entry limitations [47] and allowing for correction of user-generated content that makes unverified, negative comments [18,48] or gives inappropriate clinical advice [49].

Lack of marketing transparency is not new, with inappropriate pharmaceutical financial funding leading to US reform [50]. Similar conflicts of interest arising in digital forums require similar disclosure and transparency. Building on the Affordable Care Act [51], policymakers can expand disclosure provisions to include funding and compensation of third parties who engage in eDTCA 2.0 on behalf of pharmaceutical manufacturers. Disclosure would include recipient information, products promoted, payments, and identification of supported Web addresses, social media sites, and other eDTCA 2.0 presence. Arguably, industry online DTCA funding provided to health care professionals already falls under the Act and is subject to disclosure requirements. In any event, financial information should be prominently disclosed in eDTCA 2.0 media. Such information should be registered and placed in a public database so that consumers and policymakers have access to information about eDTCA 2.0 funding, which is an important first step in identifying its scope and impact. Concomitantly, this registration would also promote detection and shutdown of illegal online pharmacies in interactive social media.

There are potential benefits of eDTCA 2.0, similar to those identified for traditional DTCA, including motivating discussion between patients and providers, increasing patient education, and encouraging patients to seek treatment [52]. In addition, others include communication of patient safety and public health information, such as requiring manufacturer integration of patient safety information into eDTCA 2.0 by coordinating existing regulatory tools such as FDA risk evaluation and mitigation strategies, currently required to market certain products [53]. Drugs subject to risk evaluation and mitigation strategies can have this noted in eDTCA 2.0. As well, eDTCA 2.0 can integrate safety information from reliable sources, including the FDA’s MedWatch system, into online searches for health information and pharmaceutical products [8]. Access to such information could be via a link in eDTCA 2.0 tools. Furthermore, eDTCA 2.0 could be jointly used by drug regulatory authorities and pharmaceutical companies to warn about dangers associated with buying drugs online without a prescription. Official drug regulatory banners linking to the agency site with a list of approved, legitimate online pharmacies could be incorporated into such efforts, as well as public–private joint warnings about newly discovered safety issues associated with drugs [14].

Finally, regulatory efforts should provide specific manufacturer guidance as to eDTCA 2.0 limits. Although the FDA has not issued guidelines, it has held hearings discussing risk-versus-benefit reporting, manufacturer liability, and social media roles in DTCA, and announced plans to issue some guidance on the topic [8]. The FDA has only issued warning letters, including a dozen sent to pharmaceutical companies regarding online advertising deemed misbranded, but lacks a comprehensive policy [8]. More recently, Novartis was informed that its use of a Facebook media-sharing widget violated US drug marketing regulations [47]. eDTCA 2.0 regulations for companies should be clarified, but should also take into account patient privacy [54] and avoid the unintended advertising booms that followed earlier DTCA liberalization [19].

### Study Limitation

This study has several limitations. Results are descriptive and only provide a snapshot of the current state of eDTCA 2.0. The online environment is in constant flux, and findings may not reflect changes in marketing trends.

Advertising spending is likely only a rough indicator of social media presence and DTCA use. DTCA expenditures were not stratified specifically for eDTCA 2.0, since these figures are not readily available. Further, risks associated with DTCA may not be directly analogous to eDTCA 2.0 regarding product and patient safety, and can vary based on media and content.

Further, we examined only global sales revenue of the top 10 firms and products, not regional or country variations in spending or firms outside of these high sales markets. These sales data are also difficult to validate. Examination of smaller grossing firms and drugs could show different prevalence due to marketing spending limits and differing promotion strategies. We also did not validate whether sites advertising illegal online drug sales fill orders without prescriptions, since purchasing drugs over the Internet for nonmedically appropriate reasons for a fictitious patient creates legal and ethical concerns.

As well, we examined high-volume social media sites based only on popularity, but did not validate the actual number of impressions or volume of traffic on these sites. Smaller, less-visited sites may be used more by illicit drug sellers and consumers that may lead to underreporting of prevalence. Indeed, we found many social media sites of different content and origin promoting illegal online pharmacies.

Overall, DTCA globalization through eDTCA 2.0 by both drug companies and illegal online pharmacies is a global public health challenge. Licit and illicit entities have embraced the evolution in eDTCA 2.0, leaving regulatory efforts languishing. The new consumer is one that is global and connected online, a profile that precisely fits the patient/consumer of eDTCA 2.0 [55]. Public health policy must take into account this new consumer and the rapidly developing digital environment, and act to ensure that legal proscriptions against DTCA and illegal online pharmacies are followed and patient safety is protected on a global scale.

## Abbreviations

DTCA: direct-to-consumer advertising
eDTCA 2.0: direct-to-consumer advertising developed for interactive social media “Web 2.0” technologies
FDA: US Food and Drug Administration
IP: Internet protocol
RSS: really simple syndication
VIPPS: Verified Internet Pharmacy Practice Sites
